# Evidence of surface contamination in hospital rooms occupied by patients infected with monkeypox, Germany, June 2022

**DOI:** 10.2807/1560-7917.ES.2022.27.26.2200477

**Published:** 2022-06-30

**Authors:** Dominik Nörz, Susanne Pfefferle, Thomas Brehm, Gefion Franke, Ilka Grewe, Birte Knobling, Martin Aepfelbacher, Samuel Huber, Eva M. Klupp, Sabine Jordan, Marylyn M. Addo, Julian Schulze zur Wiesch, Stefan Schmiedel, Marc Lütgehetmann, Johannes K. Knobloch

**Affiliations:** * These authors contributed equally to this work and share first authorship; 1 University Medical Center Hamburg-Eppendorf (UKE), Institute of Medical Microbiology, Virology and Hygiene, Hamburg, Germany; 2 Bernhard-Nocht Institute for Tropical Medicine (BNITM), Virology Department, Hamburg, Germany; 3 University Medical Center Hamburg-Eppendorf (UKE), 1. Department of Medicine, Hamburg, Germany; 4 German Center for Infection Research, Partner site Hamburg-Lübeck-Borstel-Riems, Germany; 5 University Medical Center Hamburg-Eppendorf (UKE), Institute of Medical Microbiology, Virology and Hygiene, Department for Infection Prevention and Control, Hamburg, Germany; 6 University Medical Center Hamburg-Eppendorf (UKE), Institute for Infection Research and Vaccine Development (IIRVD), Hamburg, Germany; 7 Bernhard-Nocht Institute for Tropical Medicine (BNI), Department of Clinical Immunology of Infectious Diseases, Hamburg, Germany

**Keywords:** monkeypox virus, environmental contamination, personal protective equipment, transmission, vaccination

## Abstract

The extent of monkeypox virus environmental contamination of surfaces is unclear. We examined surfaces in rooms occupied by two monkeypox patients on their fourth hospitalisation day. Contamination with up to 10^5^ viral copies/cm^2^ on inanimate surfaces was estimated by PCR and the virus was successfully isolated from surfaces with more than 10^6^ copies. These data highlight the importance of strict adherence of hospital staff to recommended protective measures. If appropriate, pre-exposure or early post-exposure vaccination should be considered for individuals at risk.

Since 4 May 2022, the largest west-African-clade-monkeypox outbreak to date in countries with non-endemic occurrences has been described [1]. The outbreak involves transmission among people in close physical contact with symptomatic cases [1,2], in contrast to previous outbreaks, where zoonotic transmission was reported as the main mechanism of spread [3]. Nevertheless, events of person-to-person transmission have been previously described [3,4]. Additionally, transmission to personnel taking care of patients was reported on rare occasions [5,6]. Indirect transmission via contaminated objects is also discussed in the literature [6,7]. However, there are insufficient data on the environmental contamination of surfaces with monkeypox virus. We systematically examined surfaces of two hospital rooms occupied by monkeypox patients and the adjacent anterooms, which are used for donning and doffing personal protective equipment (PPE), for monkeypox virus contamination using PCR. In addition, we assessed the infectivity on cell culture of the collected samples by virus isolation.

## Sampling and virus quantification

Environmental sampling was carried out by carefully swabbing entire surfaces in the patients’ rooms and anterooms, using ESwabs, moistened with medium contained in the swab system (Copan, Brescia, Italy) on day 4 of the respective hospital stay. On larger smooth and flat surfaces, as well as on fabrics, defined areas were swabbed using sterile templates for bioburden control with cut-outs of 20 or 100 cm^2^ (SRK Collection and Transport System T2906 or T2905, Copan). In the case of a mobile phone, the entire touch screen was wiped off, since the dimensions and thus the total surface area were known. Complex structures, such as door handles, were measured and the swabbed surface area was estimated.

Samples of lesions or from the throat were also obtained from patients by swabbing with ESwabs.

Environmental or patient samples were diluted 1:1 with cobas PCR Medium (Roche, Basel, Switzerland) and monkeypox virus DNA was detected by automated real-time PCR run on the cobas 6800 system [8]. Quantification was performed using reference material (cell culture monkeypox virus DNA) quantified using digital PCR (Qiagen, Hilden, Germany) [8] resulting in digital viral copies (cp). In order to compare the various levels of contamination, the measured total viral load was calculated back to 1 cm^2^ of surface area.

Virus isolation was attempted using Vero 76 cells (ATCC CRL1587) and standard cultivation conditions for 40/50 of the collected surface specimen (Table) with 150 µL of the swab media (1 mL) as inoculates. Cultures were checked for presence of cytopathic effect (CPE) every 2 days and successful virus isolation was verified by PCR.

**Table:** Monkeypox viral load on various PCR-positive areas/objects in the isolation rooms of infected patients, given in absolute numbers and per square centimeter of surface, Germany, 22 June (n = 2 patients)

Location	Patient 1		Patient 2	
	Number of viral copies	Number of viral copies per cm^2^	Number of viral copies	Number of viral copies per cm^2^
**Patient’s room**				
Bathroom door handle, patient room side	**1.9×10^5^**	1.6×10^3^	**6.8×10^4^**	5.7×10^2^
Upper wall cabinet door handles	**1.6×10^5^**	1.3×10^3^	n. d.	n. d.
Chair seat surface	**5.8×10^4^**	5.8×10^2^	**1.4×10^5^**	1.4×10^3^
Second anteroom door, patient room side	**1.1×10^4^**	88	n. d.	n. d.
Lid of the dirty linen collection bin	**1.0×10^5^**	84	n. d.	n. d.
Intercom control buttons for staff in patient room	2.2×10^2^	11	**4.2×10^2^**	21
Base cabinet door handles	1.3×10^3^	10	n. d.	n. d.
Light switches	**6.3×10^2^**	8	**56**	2
Armrests chair	n. d.	n. d.	**1.0×10^5^**	2.1×10^2^
Window handle	n. d.	n. d.	**2.7×10^5^**	6.8×10^2^
Mobile phone touch display	n. d.	n. d.	1.5×10^4^	1.5×10^2^
Light switch bathroom	n. d.	n. d.	**3.8×10^4^**	1.5×10^3^
Handles of empty wardrobe	n. d.	n. d.	**32**	< 1
**Patient’s bathroom**				
Tap control lever	**4.8×10^6^**	2.4×10^5^	**1.1×10^5^**	5.5×10^3^
Seating surface toilet seat front in the middle	**2.5×10^6^**	1.3×10^5^	**1.5×10^4^**	7.5×10^2^
Seating surface toilet seat left	2.1×10^5^	1.0×10^4^	**2.6×10^4^**	1.3×10^3^
Seating surface toilet seat right	1.2×10^5^	5.9×10^3^	**2.6×10^4^**	1.3×10^3^
Bathroom door handle, bathroom side	**4.9×10^4^**	4.1×10^2^	**3.1×10^5^**	2.6×10^3^
Toilet flush control buttons	**6.8×10^4^**	3.4×10^2^	**1.6×10^5^**	8.0×10^2^
Soap dispenser operating lever	n. d.	n. d.	**1.7×10^6^**	4.7×10^4^
**Anteroom**				
Second anteroom door, anteroom side	**4.6×10^2^**	4	** < 10**	< 1
First anteroom door, anteroom side	**2.4×10^2^**	< 1	3.3×10^2^	3
Infectious waste garbage can handle	** < 10**	< 1	n. d.	n. d.
Disinfectant wipes lid 1	< 10	< 1	n. d.	n. d.
Disinfectant wipes lid 2	< 10	< 1	n. d.	n. d.
Switch for electronic door opener	n. d.	n. d.	** < 10**	< 1
Lid of the dirty linen collection bin	n. d.	n. d.	< 10	< 1
Handles cabinets worktop top	n. d.	n. d.	< 10	< 1
**Ward corridor**				
First anteroom door, corridor side	** < 10**	< 1	** < 10**	< 1
**Fabrics**				
Mattress cover with visible soiling	**1.7×10^6^**	1.7×10^4^	n. d.	n. d.
Comforter cover with visible soiling	**2.3×10^5^**	1.2×10^3^	n. d.	n. d.
Patient shirt middle of the bottom	**4.9×10^4^**	4.9×10^2^	n. d.	n. d.
Pillowcase without visible soiling	**6.2×10^4^**	3.1×10^2^	n. d.	n. d.
Towel in bed to protect the bed sheet	n. d.	n. d.	**1.0×10^7^**	1.0×10^5^
Pillowcase used to cover cooling packs	n. d.	n. d.	**1.6×10^6^**	8.0×10^3^
**Personal protective equipment (PPE)**				
Glove of the examiner after contact with fabrics	**3.8×10^4^**	2.7×10^2^	**1.1×10^6^**	7.9×10^3^

n. d.: not determined.

Samples for which virus cultivation on cell cultures was attempted are indicated by bold font. Samples with successful virus isolation are marked in grey. Numbers of viral copies ≤ 100 in magnitude and respective values per cm^2^ are presented as round numbers.

## Patient characteristics

Both monkeypox patients were men in their thirties. In patient 1, skin lesions – erythema, pustules, excoriations with crustae – were present in the anus, perianal region and scrotum, penis, and a few isolated lesions on the legs, trunk, tongue, and buccal mucosa. In patient 2, lesions were only present in the anus and the perianal region. The highest viral loads in the patients’ specimens were observed in lesional swabs with a maximum of 2.7×10^8^ and 4.4×10^8^ cp for patients 1 and 2, respectively. In throat swab samples a respective maximum of 1.3×10^6^ and 2.1×10^7^ cp was detected.

## Monkeypox virus contamination

Both patients were hospitalised in isolation rooms with an associated bathroom. The patients’ rooms were separated from the ward corridor by anterooms, which were used for donning and doffing PPE (Figure, exemplary for patient 1).

**FIGURE:**
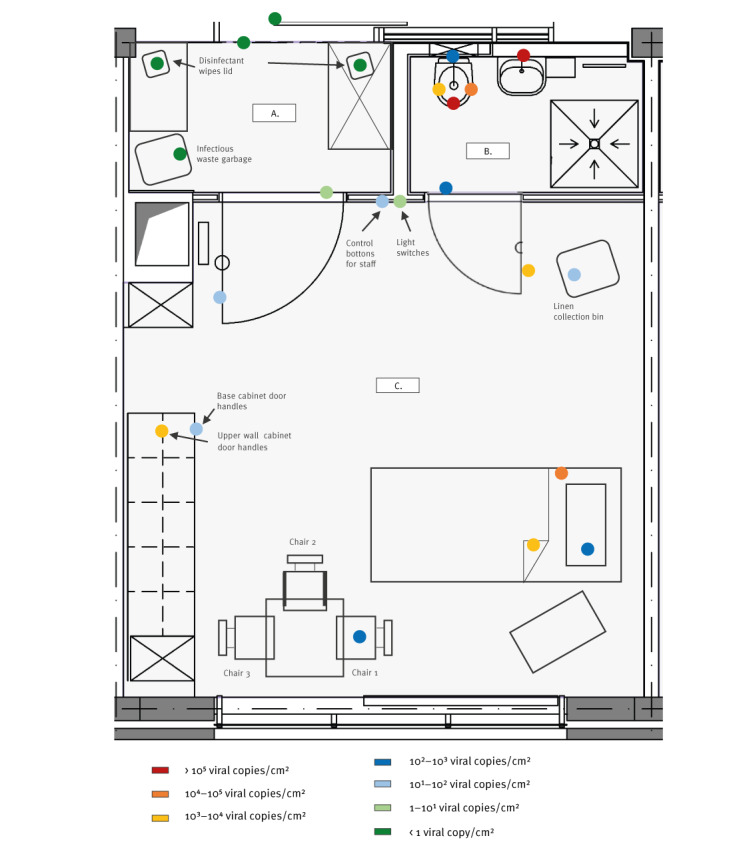
Outline map of the (A) anteroom (B) bathroom and (C) room of a hospitalised patient^a^ infected with monkeypox virus, with various sampled-surface locations and measured monkeypox virus contamination levels, Germany, June 2022

^a ^The room of patient 1 is depicted as an example.

The coloured disks are indicative of the amounts of viral copies per surface area found in different locations of the patient’s room, bathroom and anteroom. Each colour corresponds to a range, or maximum/minimum threshold, of viral genome copies per cm^2^.

All surfaces directly touched by the patients’ hands showed viral contamination with the highest loads detected in both bathrooms. A value of 2.4×10^5^ cp/cm^2^ was obtained for the tap control lever of the wash basin of patient 1; for the operating lever of the soap dispenser of patient 2, the value was 4.7×10^4^ cp/cm^2^ (Table). Similarly, high viral loads (1.3×10^5^ and 1.3×10^3^ cp/cm^2 ^for bathrooms of patients 1 and 2, respectively) were detected on toilet seats. Seat surfaces of chairs, which patients reported using most frequently also showed up to 1.4×10^3^ cp/cm^2^. On the touch display of the mobile phone of patient 2, a total of 1.5×10^2^ cp/cm^2^ was observed.

Monkeypox virus DNA was also found on the patients' room surfaces, presumably touched primarily by medical personnel. The highest level (1.3×10^3^ cp/cm^2^) was found on upper wall cabinet door handles in the room of patient 1. Viral DNA was observed on all other investigated surfaces in the patients’ rooms, although it was not known at the time of testing whether and to what extent the patients had also touched these surfaces.

Fabrics that were extensively used by the patients also showed viral contamination up to 10^5^ cp/cm^2^ (Table). Immediately after handling the fabrics, the palmar side of the investigator's right gloved hand was swabbed and confirmed to be contaminated in investigations related to both patient’s rooms (2.7×10^2^ and 7.9×10^3^ cp/cm^2^). Interestingly, we were able to demonstrate infectivity to Vero 76 cells by successful virus isolation for three of the collected samples relative to patient 2, namely the investigator's glove, the soap dispenser operating lever, and a towel on the patient’s bed (Table). All three samples had more than 10^6^ copies per sample (>10^3^ cp/cm^2^).

In the anteroom, all hand-contact points examined yielded positive PCR results. However, only traces of viral DNA (maximum = 3 cp/cm^2^) were detected on the handle of the door leading to the patient's room. Traces of viral DNA were identified on the handle of both anteroom doors located in the ward corridor, outside the anteroom.

## Discussion

Besides zoonotic transmission, monkeypox virus infections have been reported after person-to-person transmission [3]. To our knowledge, the highest rate of secondary cases described to date was in a central African outbreak in 1996–1997, where 65 (73%) of 89 case-patients with available data had had contact to another case-patient within 7–21 days before their onset of illness [4]. Person-to-person transmission with nosocomial transmission from a patient to three healthcare workers was reported in another African outbreak [5]. One nurse who evaluated the patient, and who later became ill, had removed the patient’s clothing, taken the patient’s temperature, and drawn blood without adequate PPE. Nosocomial transmission was also reported related to an imported case from Nigeria to the United Kingdom [6]. In this case, the infected healthcare worker changed potentially contaminated bed linen without adequate PPE.

There are no definite data on the required infectious dose with monkeypox virus in humans. However, in contrast to variola virus [9], a significantly higher dose is assumed to be required to trigger infection [10]. In non-human primates, infection could be initiated by intrabronchial application of 5×10^4^ plaque-forming units (PFU) [11]. Orthopoxviruses are reported to remain infectious under dry conditions and different temperatures [12]. Dried vaccinia virus is stable up to 35 weeks (at 4 °C) without loss of infectivity [12]. In this study monkeypox virus was successfully isolated from three different samples, each with a total of at least 10^6^ virus copies. Thus, contaminated surfaces with such viral loads or higher, could potentially be infectious and it cannot be ruled out that their contact with especially damaged skin or mucous membranes, could result in transmission.

Detection of up to 1.1×10^6^ viral copies on gloves is consistent with the detection of viral DNA on surfaces typically handled only by medical staff such as the door handles of the anteroom. The detection of the virus at very low concentrations even outside the isolation unit indicates that containment protocols may not have been fully adhered to.

The findings in this report are subject to some limitations. As DNA is an environmentally stable molecule, detection of viral DNA by PCR cannot be equated with infectious virus. Despite high contamination with up to 10^5^ cp/cm^2^ as well as the successful recovery of monkeypox virus from samples with a total of > 10^6^ copies, our findings do not prove that infection can occur from contact with these surfaces. No secondary case in the context of clinical care of the two patients in our study has been observed so far. The study was performed only for two cases and might not be generalised to other cases. In particular, in certain cases, depending on the skin regions mainly affected and the number of lesions, the levels of contamination of different surfaces may vary. 

Overall, these data underscore the importance to remind hospital personnel of the need to follow recommended protection measures for monkeypox. Sufficient time and attention must be given to the careful doffing of PPE and personnel must be properly trained in these procedures. Regular disinfection of frequent hand and skin contact points during the care processes additional to regular room cleaning and surface disinfection using products with at least virucidal activity against enveloped viruses can reduce infectious virus on surfaces and thereby risk of nosocomial transmission [13]. Suitable strategies for preventing the spread of the virus outside the patient's room must be individually adapted to the situation of the respective medical facility. The application of the double-gloving method [14] with discarding of the outer glove layer or disinfection of the gloved hand [15,16] before entering an anteroom can contribute to this. After the final doffing of the PPE, proper hand hygiene must be performed immediately. Pre-exposure vaccination for healthcare workers [17,18] as well as early post-exposure vaccination in the case of probable or confirmed contamination in the absence of or incorrectly applied protective equipment [18,19] may be considered. 

Those living in the same households of affected individuals should be advised that, in addition to avoiding close physical contact, disinfection of shared skin- and hand-contact surfaces might be useful to prevent transmission [20,21]. At the present time, the viral load on inanimate surfaces required for disease transmission is unknown. Therefore, future studies should also investigate the dose dependent infectivity of such surfaces.
